# Chest CT–derived pulmonary artery enlargement at the admission predicts overall survival in COVID-19 patients: insight from 1461 consecutive patients in Italy

**DOI:** 10.1007/s00330-020-07622-x

**Published:** 2020-12-23

**Authors:** Antonio Esposito, Anna Palmisano, Marco Toselli, Davide Vignale, Alberto Cereda, Paola Maria Vittoria Rancoita, Riccardo Leone, Valeria Nicoletti, Chiara Gnasso, Alberto Monello, Andrea Biagi, Piergiorgio Turchio, Giovanni Landoni, Guglielmo Gallone, Giacomo Monti, Gianni Casella, Gianmarco Iannopollo, Tommaso Nannini, Gianluigi Patelli, Luisa Di Mare, Marco Loffi, Pietro Sergio, Davide Ippolito, Sandro Sironi, Gianluca Pontone, Daniele Andreini, Elisabetta Maria Mancini, Clelia Di Serio, Francesco De Cobelli, Fabio Ciceri, Alberto Zangrillo, Antonio Colombo, Carlo Tacchetti, Francesco Giannini

**Affiliations:** 1 Experimental Imaging Center, IRCCS San Raffaele Scientific Institute, Via Olgettina 60, Milan, Italy; 2 School of Medicine, Vita-Salute San Raffaele University, Via Olgettina 58, Milan, Italy; 3 GVM Care & Research Maria Cecilia Hospital, Cotignola, Italy; 4 Centro Universitario di Statistica per le Scienze Biomediche, Vita-Salute San Raffaele University, Milan, Italy; 5 Guglielmo da Saliceto Hospital, Piacenza, Italy; 6 Anesthesia and Intensive Care Department, IRCCS San Raffaele Scientific Institute, Milan, Italy; 7 Division of Cardiology, Department of Internal Medicine, Città della Salute e della Scienza, Turin, Italy; 8 Ospedale Maggiore, Bologna, Italy; 9 ASST Bolognini Hospital, Bergamo Est, Italy; 10 Ospedale di Cremona, Cremona, Italy; 11 San Gerardo Hospital, Monza, Italy; 12 ASST Papa Giovanni XXIII, Bergamo, Italy; 13 Centro Cardiologico Monzino IRCCS, Milan, Italy; 14 Department of Hematology and Bone Marrow Transplantation, IRCCS San Raffaele Scientific Institute, Milan, Italy

**Keywords:** COVID-19, Thorax, Tomography, X-ray computed, Hypertension, pulmonary, Pulmonary artery

## Abstract

**Objectives:**

Enlarged main pulmonary artery diameter (MPAD) resulted to be associated with pulmonary hypertension and mortality in a non-COVID-19 setting. The aim was to investigate and validate the association between MPAD enlargement and overall survival in COVID-19 patients.

**Methods:**

This is a cohort study on 1469 consecutive COVID-19 patients submitted to chest CT within 72 h from admission in seven tertiary level hospitals in Northern Italy, between March 1 and April 20, 2020. Derivation cohort (*n* = 761) included patients from the first three participating hospitals; validation cohort (*n* = 633) included patients from the remaining hospitals. CT images were centrally analyzed in a core-lab blinded to clinical data. The prognostic value of MPAD on overall survival was evaluated at adjusted and multivariable Cox’s regression analysis on the derivation cohort. The final multivariable model was tested on the validation cohort.

**Results:**

In the derivation cohort, the median age was 69 (IQR, 58–77) years and 537 (70.6%) were males. In the validation cohort, the median age was 69 (IQR, 59–77) years with 421 (66.5%) males. Enlarged MPAD (≥ 31 mm) was a predictor of mortality at adjusted (hazard ratio, HR [95%CI]: 1.741 [1.253–2.418], *p* < 0.001) and multivariable regression analysis (HR [95%CI]: 1.592 [1.154–2.196], *p* = 0.005), together with male gender, old age, high creatinine, low well-aerated lung volume, and high pneumonia extension (c-index [95%CI] = 0.826 [0.796–0.851]). Model discrimination was confirmed on the validation cohort (c-index [95%CI] = 0.789 [0.758–0.823]), also using CT measurements from a second reader (c-index [95%CI] = 0.790 [0.753;0.825]).

**Conclusion:**

Enlarged MPAD (≥ 31 mm) at admitting chest CT is an independent predictor of mortality in COVID-19.

**Key Points:**

**•** *Enlargement of main pulmonary artery diameter at chest CT performed within 72 h from the admission was associated with a higher rate of in-hospital mortality in COVID-19 patients.*

**•** *Enlargement of main pulmonary artery diameter (≥ 31 mm) was an independent predictor of death in COVID-19 patients at adjusted and multivariable regression analysis.*

**•** *The combined evaluation of clinical findings, lung CT features, and main pulmonary artery diameter may be useful for risk stratification in COVID-19 patients.*

**Supplementary Information:**

The online version contains supplementary material available at 10.1007/s00330-020-07622-x.

## Introduction

The coronavirus disease 2019 (COVID-19) rapidly invaded the world affecting millions of people and becoming a global health emergency.

Sparse autopsies on patients with COVID-19 have found interstitial pneumonia with diffuse alveolar damage, pulmonary arterioles thrombosis [1], and right ventricle (RV) dilation [2], suggesting that increased RV afterload due to endothelial injury [3] with lung vessel micro-thrombosis [4] might be a pathological driver in critical COVID-19 illness.

In two recent studies [5, 6] on 120 and 115 COVID-19 patients undergoing echocardiography, non-survivors displayed larger right-heart chambers, reduced RV function, and elevated pulmonary artery systolic pressure compared to survivors. In light of the possible impact of RV afterload on patients’ outcome and treatment [7–9], more robust data coming from larger populations are needed to strengthen the current evidence. Data from echocardiography are scarce, because of technical [5] and practical limitations, mainly related to limited resources in a scenario of health emergency and to the need of reducing healthcare workers’ exposure.

Chest CT has been widely adopted for COVID-19 pneumonia diagnosis [10], monitoring, and prognostication [11]. Beyond the extraction of parameters describing the features of lung involvement and its severity, the main pulmonary artery diameter (MPAD) can be easily measured from a chest CT scan. Enlarged MPAD on CT has been studied as a method for the screening and diagnosis of pulmonary hypertension (PH) [12–15]. Reference values for healthy patients have been previously established [16] and showed excellent sensitivity for excluding PH [13]. In the pre-COVID-19 era, enlargement of MPAD resulted to be associated with PH severity at RV catheterization and to higher rate of mortality [13]. Hence, we hypothesized that the measurement of MPAD, a known marker of pulmonary hypertension, could be useful for risk stratification of COVID-19 patients.

The aim of the present study was to evaluate the prognostic value of MPAD enlargement at the admission chest CT on overall survival in COVID-19 patients considering a multivariable setting, and to validate the final multivariable model.

## Materials and methods

### Study setting and participants

This is a multicenter retrospective cohort study. Study participants were recruited among all adult patients (age 18 years or older) with confirmed RT-PCR for SARS-CoV-2, submitted to chest CT within 72 h from admission in seven tertiary level hospitals located in Northern Italy ((*1) IRCCS San Raffaele Scientific Institute, Milan (OSR); (2) Guglielmo da Saliceto Hospital, Piacenza (PCZ); (3) Ospedale Maggiore, Bologna (BOL); (4) ASST Bolognini Hospital Bergamo Est (BGE); (5) Ospedale di Cremona (CRE); (6) San Gerardo Hospital, Monza (SGM); (7) Centro Cardiologico Monzino IRCCS, Milano (CCM*)), between March 1 and April 20, 2020. Missing data on comorbidities or follow-up were considered exclusion criteria. The study was approved by the local ethics committees and written informed consent was obtained.

Clinical data were collected by each center according to a centralized electronic case report form (CRF). Clinical data at the admission consisted of the following: demographic characteristics (sex and age), comorbidities (hypertension, diabetes, chronic lung disease, cardiovascular disease), and laboratory tests: white blood cell count (WBC), creatinine, C-reactive protein (CRP), lactate dehydrogenase (LDH), troponin I, interleukin-6, and D-dimer. Outcome data were oro-tracheal intubation and death.

All chest CT images were collected and analyzed in a single core-lab (*Experimental Imaging Center, IRCCS San Raffaele Scientific Institute, Milan, Italy*) blinded to clinical data.

The derivation cohort consisted of patients from the three hospitals (OSR, PCZ, BOL) that completed the CRF and shared chest CT images for centralized analysis within May 20, 2020, established as the first deadline for data collection. Patients from the remaining four participating hospitals (BGE, CRE, SGM, CCM), who provided data and CT images within the end of June, were used for the external validation. Prior to the analysis, data were cross-checked with medical charts and verified by data managers and clinicians for accuracy.

**Fig. 1:**
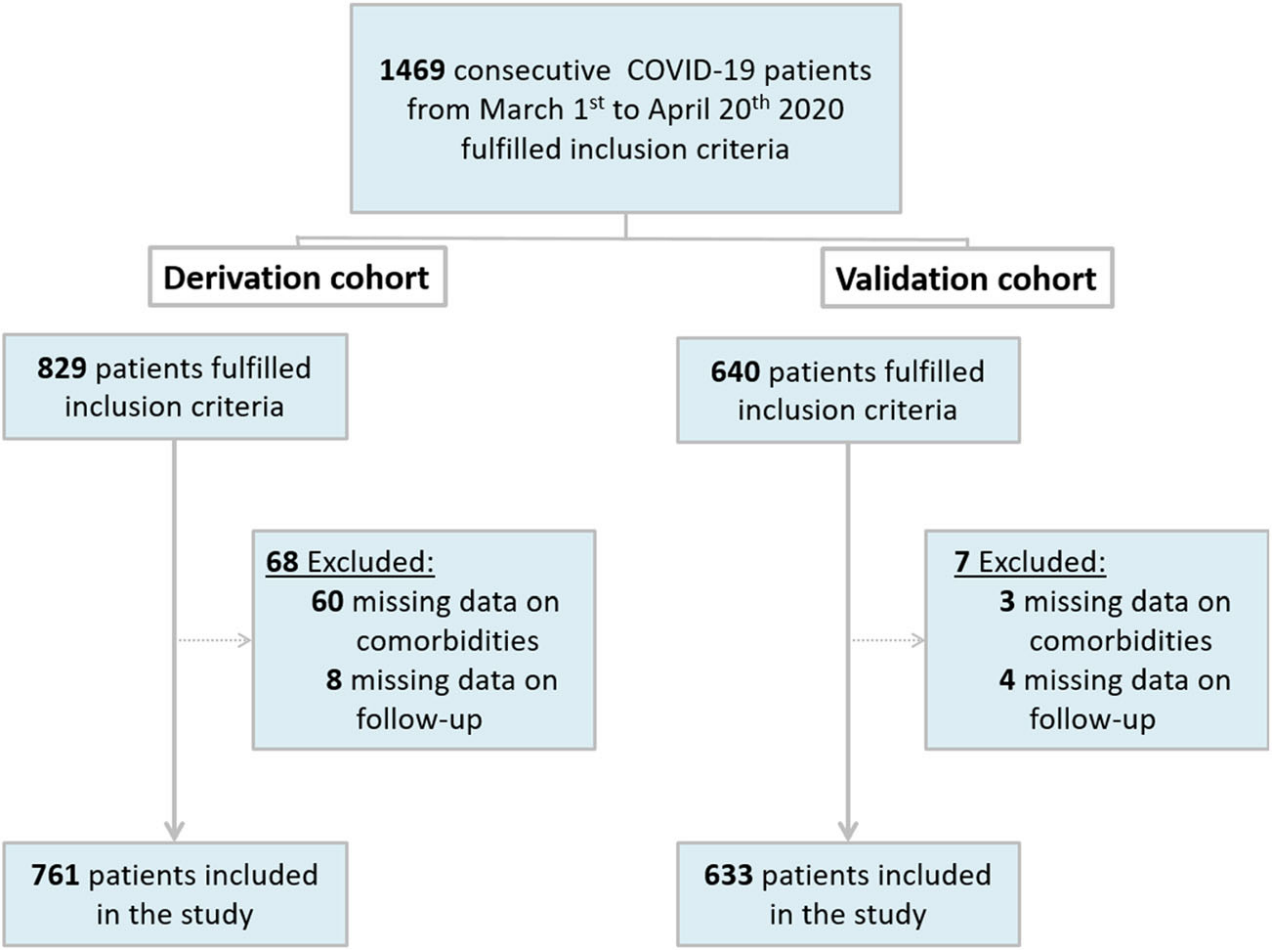
Enrollment flowchart

A total of 1469 consecutive patients fulfilled the inclusion criteria: 68 of the 829 patients of the derivation cohort and 7 of the 640 patients of the validation cohort were excluded for lacking data on comorbidities or follow-up (Fig. 1). The median follow-up was 51 days for the derivation cohort and 63 days for the validation cohort.

Complete demographic and clinical features of both cohorts are presented in Table 1.

**Table 1:** Patient characteristics

	Derivation cohort		Validation cohort	
Variables at the admission	*N*	Median [IQR]	*N*	Median [IQR]
Age, years	761	69.25 [58.01–76.87]	633	69.14 [59.03–77.43]
Male	761	537 (70.6%)	633	421 (66.5%)
Hypertension, no. (%)	761	484 (63.6%)	633	285 (45%)
Diabetes, no. (%)	761	150 (19.7%)	633	119 (18.8%)
Known cardiovascular disease, no. (%)	761	95 (12.5%)	633	129 (20.4%)
Known chronic lung disease, no. (%)	761	95 (12.5%)	633	44 (7%)
Oxygen saturation, %	684	92 [88–95]	571	92 [88–96]
White blood cell count/mm^3^	761	6655 [5000–9522.5]	628	7025 [5087.5–9577.5]
Creatinine, mg/dL	761	1.01 [0.83–1.29]	625	1.01 [0.84–1.28]
LDH, U/L	717	368 [275–491]	240	320 [230.25–464.25]
CRP, mg/dL	761	11.3 [5.62–19.21]	615	9.7 [3.57–17.23]
Troponin I, ng/L	144	13.65 [6.07–43.4]	51	18 [10.5–80.5]
IL-6, pg/mL	165	38.1 [17.6–104]	96	100.1 [32.79–495.05]
D-dimer, mcg/mL	232	1.54 [0.59–4.29]	223	1.9 [0.72–4]
Main pulmonary artery diameter, mm	761	27 [25–29]	633	27 [25–30]
Main pulmonary diameter, no. (%)	761		633	
Normal		541 (71.1%)		441 (69.7%)
Mildly enlarged		86 (11.3%)		58 (9.2%)
Moderately enlarged		98 (12.9%)		101 (15.9%)
Severely enlarged		36 (4.7%)		33 (5.2%)
Left pulmonary artery diameter, mm	761	20 [18–22]	633	21 [19–23]
Right pulmonary artery diameter, mm	761	21 [19–23]	633	21 [19–23]
Well-aerated lung volume, cm^3^	761	2302 [1332–3419]	633	2283 [1383–3374]
GGO/consolidation ratio, no. (%)	761		633	
0		9 (1.2%)		7 (1.1%)
1		432 (56.8%)		328 (51.8%)
2		145 (19.1%)		142 (22.4%)
3		175 (23%)		156 (24.6%)
Pneumonia score, no. (%)	761		633	
0		9 (1.2%)		7 (1.1%)
1		250 (32.9%)		181 (28.6%)
2		363 (47.7%)		249 (39.3%)
3		130 (17.1%)		151 (23.9%)
4		9 (1.2%)		45 (7.1%)
Outcome				
Non-survivors, no. (%)	761	182 (23.9%)	633	140 (22.1%)

Data are reported as median [interquartile range, IQR], except otherwise specified

*LDH*, lactate dehydrogenase; *CRP*, C-reactive protein; *IL-6*, interleukin 6; *GGO*, ground-glass opacity

### Outcomes

The primary outcome was overall survival (OS) measured from admission to the emergency department. Secondary outcomes were in-hospital mortality and time-to-orotracheal intubation from admission to the emergency department.

### Chest CT scan

All chest CT examinations were performed on multidetector scanners with at least 16 detector rows. The list of scanners and acquisition protocols are reported in Supplementary Materials (ESM 1). Briefly, all volumetric chest scans were sent to the core-lab where they were reformatted at a 2.5-mm slice thickness without gap or overlap. Images were reconstructed with sharp kernel for lung parenchyma evaluation and a soft kernel for mediastinum evaluation, and they were visualized using a standard window (lung: width 1400 HU, center − 450 HU; mediastinum: width 350 HU, center 40 HU)**.**

### Chest CT image analysis

**Fig. 2:**
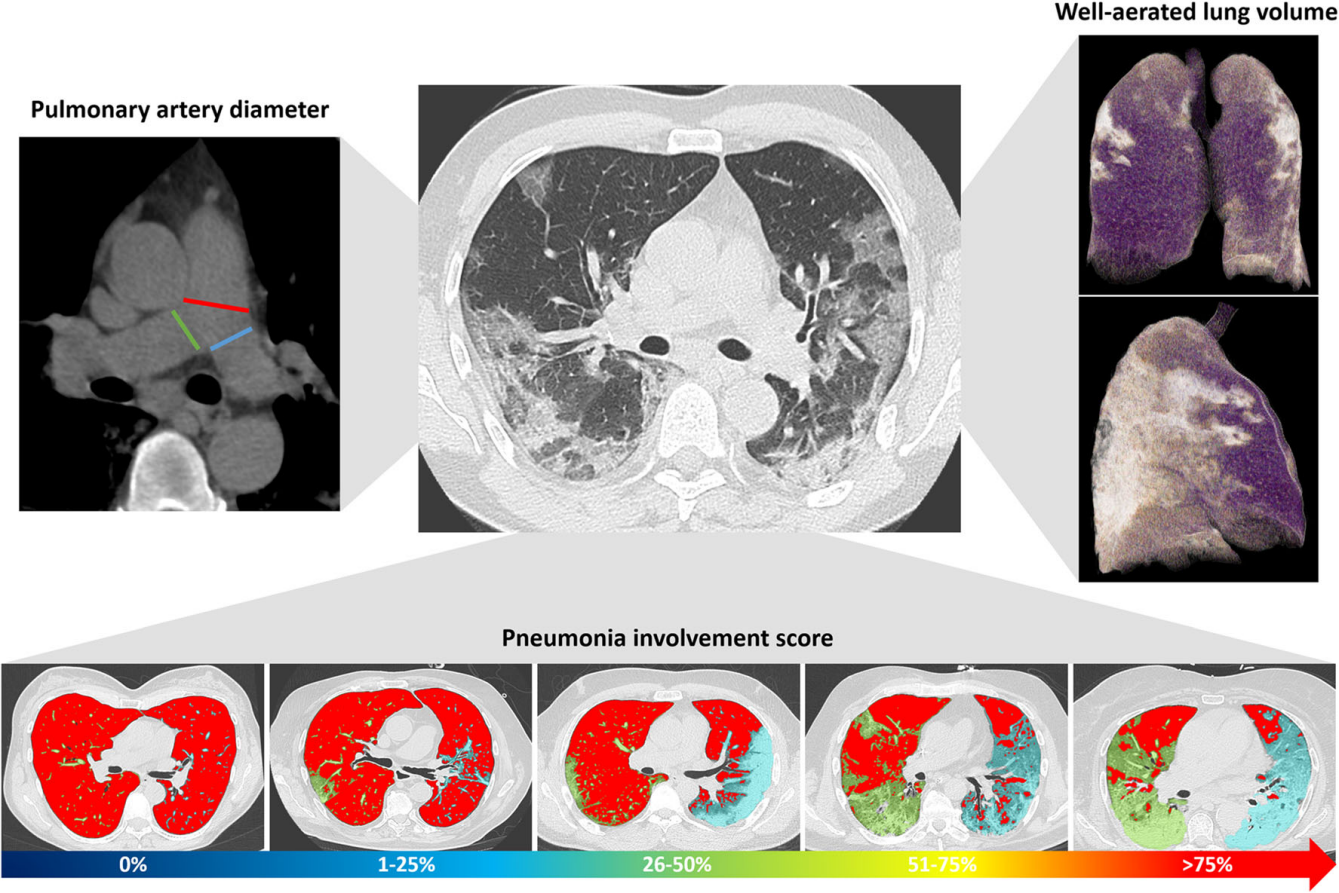
Imaging parameters extracted from chest CT in COVID-19 patients. For each patient, the diameter of the main pulmonary artery (red line) was measured at the level of its bifurcation and the diameter of right and left pulmonary arteries at the level of their origin (green and blue lines, respectively) on the mediastinal window reconstruction (top left of the panel). Using dedicated software, well-aerated lung volume was automatically extracted (violet parenchyma in 3D lung volume rendering on the top right of the panel). Pneumonia involvement was scored from 0 to 4 (score 0: 0%; score 1: 1–25%; score 2: 26–50%; score 3: 51–75% score 4: > 75%); well-aerated lung parenchyma is displayed in red, and pneumonia in green for the right lung and in blue for the left lung (bottom of the panel)

The analysis was performed by a radiologist with 9 years of experience in cardiothoracic imaging, blinded to all clinical data in order to reduce the possible bias in the analysis and to increase homogeneity and reliability of data. Parameters of lung involvement (residual respiratory lung reserve, pneumonia extension, and features) and pulmonary artery metrics were extracted (Fig. 2).

Residual respiratory lung reserve was measured as well-aerated lung volume, quantified using automatic software (IntelliSpace Portal 8.0, Philips Medical Systems) based on a previously defined HU threshold value [17]. Pneumonia extension was scored in 0% (absent, score 0), 1–25% (minimal, score 1); 26–50% (mild, score 2); 51–75% (moderate, score 3); and > 75% (severe, score 4). Qualitative features of pneumonia were scored as follows: score 0 for absent pneumonia, score 1 for prevalent ground-glass opacities (GGOs), score 2 for prevalent consolidation, and score 3 for GGOs and consolidation equally represented. The metrics of pulmonary arteries included the measurement of the MPAD at the level of its bifurcation, of the left (LPAD) and of the right pulmonary artery diameter (RPAD) at their origin. MPAD was classified according to the severity classification system for the diagnosis and prognosis of PH [13] in four classes: normal (≤ 27 mm for females and ≤ 29 mm for males, according to the Framingham sex-specific normative values [16]); mild enlargement (from > 27 to < 31 mm for females and > 29 to < 31 mm for males); moderate enlargement (from ≥ 31 to 34 mm for both sexes), and severe enlargement (> 34 mm for both sexes). To assess inter-observer reliability, a second reader with 5 years of experience in cardiothoracic imaging, blinded to the measurements of the first reader, assessed MPAD and pneumonia score.

### Statistical analysis

Comparisons between numerical variables were performed with the Mann-Whitney test, and comparisons between categorical variables with Fisher’s exact test. *P* values were adjusted with Bonferroni’s correction to account for multiple testing. Follow-up data were censored at 40 days from hospital admission. OS was estimated with the Kaplan-Meier estimator and groups were compared with the log-rank test. The cumulative incidence of orotracheal intubation was estimated using the competing risk approach (with death without intubation as a competing event) and groups were compared with Gray’s test.

Cox’s regression analysis was employed for evaluating the role of MPAD classes [13, 16] in predicting the OS, un-adjusting and adjusting for demographics and comorbidities (age, sex, hypertension, diabetes, history of cardiovascular disease, and chronic lung disease). Multivariable Cox’s regression analysis was employed for evaluating the simultaneous role of CT parameters (MPAD, pneumonia extension, well-aerated lung volume), laboratory tests (WBC, CRP, creatinine), demographics, and comorbidities in predicting OS. The final model was obtained with a backward variable selection with a removal significant level of 0.05. Model calibration was performed as detailed in Supplementary Materials (ESM 1). Model discrimination was evaluated with the optimism corrected c-index. To assess the role of gender in categorized MPAD with respect to the outcome, the final Cox model was estimated adding an interaction term between the two variables.

The adjusted Cox regression analysis, the calibration curve, and the c-index of the final multivariable Cox’s model were computed on the validation cohort using CT data from two independent readers.

Inter-observer reliability was assessed with Rothery’s non-parametric intra-class correlation coefficient (ICC) [18] for MPAD and Cohen’s kappa coefficient for the pneumonia score.

Missing data were not imputed; thus, each analysis considered only complete cases for the variables used in the analysis. All tests were 2-sided and the significance level was set at 0.05. Confidence intervals (CIs) were computed at a 95% level. All statistical analyses were performed using R 3.5.0 (http://www.R-project.org/). All details are reported in Supplementary Materials (ESM 1).

## Results

### Derivation cohort: clinical and lung CT characteristics

The median age was 69 (interquartile range IQR, 58–77) years and 537 (70.6%) patients were male (Table 1). Hypertension was the most frequent comorbidity (484 patients, 63.6%) and median oxygen saturation was 92% (IQR, 88–95%).

At chest CT, 752 patients (98.8%) had pneumonia, with prevalent GGOs (432 patients, 56.8%). Pneumonia extension greater than 50% was recorded in 139 cases (18.3%).

Median MPAD was 27 [25–29] mm. Twenty-one (2.8%) patients were not hospitalized after emergency department evaluation, while 459 (60.3%) patients were discharged within 40 days from admission with a median hospitalization length of 11 (IQR, 7–17) days. The cumulative incidence of orotracheal intubation at 40 days was 21.28%. At 40 days, non-survivors were 182/761 patients (23.9%), with death occurring at a median time from an admission of 7 (IQR, 4–12) days. The characteristics of survivors and non-survivors are reported in Table 2. OS at 40 days was 76.08% (95% CI: 73.11–79.17%).

**Table 2:** Clinical and CT features of survivors vs non-survivors in the derivation cohort

	Survivors (40-days FU)		Non-survivors (40-days FU)		
Variables	*N*	Median [IQR]	*N*	Median [IQR]	adj. *p* value
Age, years	579	64.9 [55.66–73.96]	182	76.97 [71.37–82.49]	< 0.001
Male	579	396 (68.4%)	182	141 (77.5%)	0.396
Hypertension, no. (%)	579	340 (58.7%)	182	144 (79.1%)	< 0.001
Diabetes, no. (%)	579	99 (17.1%)	182	51 (28%)	0.037
Known cardiovascular disease, no. (%)	579	58 (10%)	182	37 (20.3%)	0.009
Known chronic lung disease, no. (%)	579	60 (10.4%)	182	35 (19.2%)	0.057
Oxygen saturation, %	579	93 [90–96]	182	88 [83–92]	< 0.001
White blood cell count/mm^3^	579	6400 [4900–9100]	182	7250 [5340–11,020]	0.051
Creatinine, mg/dL	579	0.97 [0.8–1.19]	182	1.26 [1–1.78]	< 0.001
LDH, U/L	553	347 [258–449]	164	462.5 [354.5–618.5]	< 0.001
CRP, mg/dL	579	10.4 [5.07–18.18]	182	13.96 [8.43–21.12]	0.003
Troponin I, ng/L	125	12.1 [5.4–33.5]	19	76.2 [49.7–514.1]	< 0.001
IL-6, pg/mL	150	33.8 [14.77–92.02]	15	124 [69.2–240]	0.011
D-dimer, mcg/mL	203	1.32 [0.59–3.88]	29	3.92 [0.88–9.22]	0.445
Main pulmonary artery diameter, mm	579	27 [24–29]	182	28 [26–31]	< 0.001
Left pulmonary artery diameter, mm	579	20 [18–22]	182	21.3 [20–24]	< 0.001
Right pulmonary artery diameter, mm	579	20 [18–23]	182	22 [20–25]	< 0.001
Well-aerated lung volume, cm^3^	579	2506 [1564–3609]	182	1520.5 [1009–2544]	< 0.001
GGO/consolidation ratio, no. (%)	579		182		1.000
0		9 (1.6%)		0 (0%)	
1		318 (54.9%)		114 (62.6%)	
2		117 (20.2%)		28 (15.4%)	
3		135 (23.3%)		40 (22%)	
Pneumonia score, no. (%)	579		182		< 0.001
0		9 (1.6%)		0 (0%)	
1		220 (38%)		30 (16.5%)	
2		268 (46.3%)		95 (52.2%)	
3		77 (13.3%)		53 (29.1%)	
4		5 (0.9%)		4 (2.2%)	

Data are reported as median [IQR], except otherwise specified

*IQR*, interquartile range; *adj. p value*, adjusted *p* value with Bonferroni’s correction; *LDH*, lactate dehydrogenase; *CRP*, C-reactive protein; *IL-6*, interleukin 6; *GGO*, ground-glass opacity

### Derivation cohort: pulmonary enlargement and mortality

Out of 761 patients, 86 (11.3%) had mild enlargement, 98 (12.9%) moderate enlargement, and 36 (4.7%) had severe MPAD enlargement.

The cumulative incidence of orotracheal intubation was not significantly different among the classes of MPAD, despite a numerically higher incidence in patients with a severe enlargement (at 40 days [95% CI]: for normal 21.44% [18.09–24.99%], for mild enlargement 17.44% [10.27–26.19%], for moderate enlargement 21.43% [13.91–30.02%], and for severe enlargement 27.78% [14.34–42.96%]; Gray’s test *p* = 0.609).

**Fig. 3:**
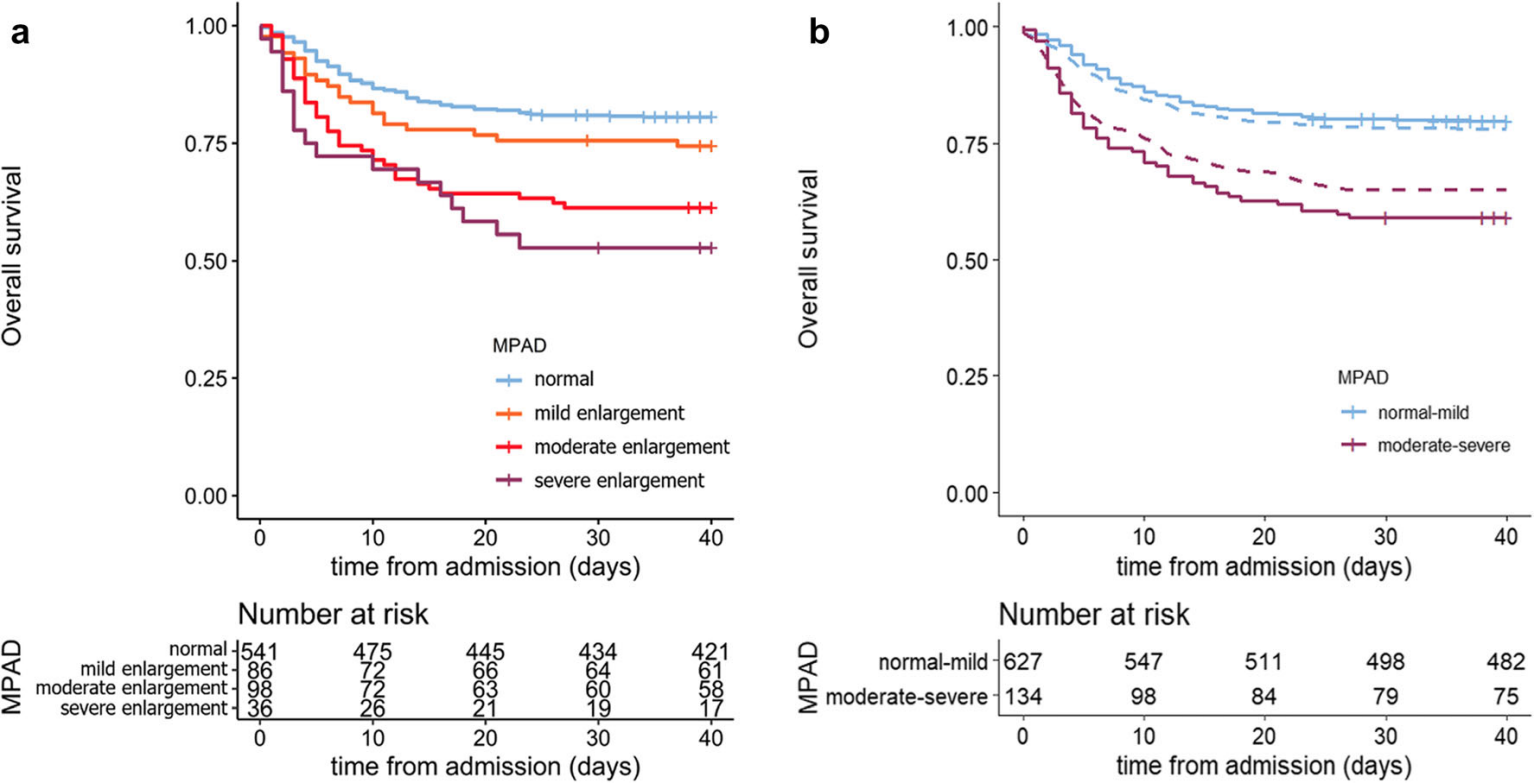
Overall survival in COVID-19 patients according to four-tier (**a**) and two-tier (**b**) classification of main pulmonary artery enlargement. The Kaplan-Meier curves show the overall survival in COVID-19 patients according to the four-tier classification system of MPAD classification (**a**) [13]. The four classes of MPAD were associated with a different risk of death (40-day overall survival [95% CI]: 80.56% [77.32–83.99%] for normal MPAD, 74.40% [65.72–84.23%] for mild enlargement, 61.22% [52.30–71.67%] for moderate enlargement, 52.78% [38.75–71.89%] for severe enlargement; log-rank test *p* < 0.001). In **b** are reported the Kaplan-Meier curves (solid lines) and the adjusted Kaplan-Meier curves (dashed lines) with inverse probability weighting based on propensity score (detailed in Supplementary Materials ESM 1) showing the overall survival in COVID-19 patients according to two-tier MPAD enlargement (moderate-severe vs normal-mild). The two classes of MPAD enlargement were associated with a different risk of death (40-day OS [95% CI]: 79.74% [76.65–82.95%] for normal-mild enlargement MPAD and 58.96% [51.19–67.90%] for moderate-severe enlargement; log-rank test *p* < 0.001; adjusted log-rank test with inverse probability weighting based on propensity score *p* = 0.002)

The mortality rate was 25.6% (22/86), 38.8% (38/98), and 47.2% (17/36) in patients with mild, moderate, and severe MPAD enlargement, respectively, while it was 19.4% (105/541) in patients with normal MPAD (global Fisher’s exact test *p* < 0.001). Indeed, the classes of MPAD were associated with a different risk of death (40-day overall survival [95% CI]: for normal 80.56% [77.32–83.99%], for mild enlargement 74.40%[65.72–84.23%], for moderate enlargement 61.22% [52.30–71.67%], and for severe enlargement 52.78% [38.75–71.89%]; log-rank test *p* < 0.001) (Fig. 3a). At Cox’s regression analysis, moderate to severe MPAD enlargement (≥ 31 mm) resulted to predict a significantly reduced overall survival with respect to normal MPAD (hazard ratio, HR [95% CI]: mild enlargement vs normal 1.387 [0.876–2.196], *p* = 0.163; moderate enlargement vs normal 2.311 [1.594–3.350], *p* < 0.001; severe enlargement vs normal 3.001 [1.797–5.010], *p* < 0.001). Similar results were obtained also at unadjusted Cox’s regression analysis (Table S1). Therefore, in the subsequent analyses, the MPAD was dichotomized into moderate-severe MPAD enlargement (≥ 31 mm) vs normal-mild MPAD enlargement (< 31 mm). The percentage of moderate-severe MPAD resulted to be similar between genders (M: 18.6% vs F: 15.2%, *p* = 1) (Table S2). The new categorization achieved HR = 1.741 (95% CI: 1.253–2.418, *p* < 0.001) at adjusted Cox’s regression (at unadjusted analysis: HR = 2.367 [1.725; 3.249], *p* < 0.001) (Fig. 3b).

**Fig. 4:**
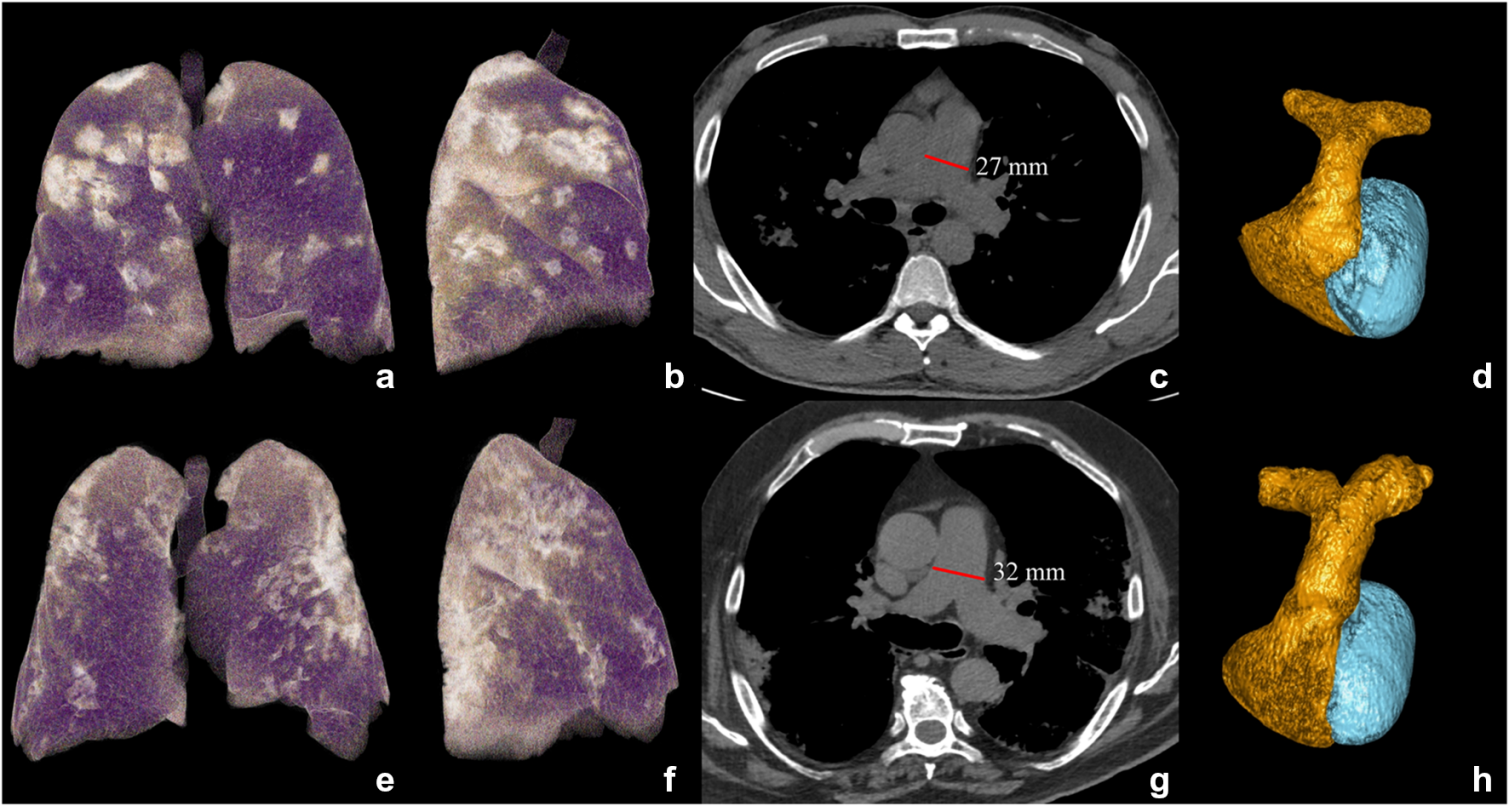
Exemplifying cases: a survivor (top) and non-survivor (bottom) patients. CT 3D volume rendering of lungs in antero-posterior view (**a**, **e**) and lateral view (**b**, **f**), CT axial images with mediastinal window (**c**, **g**) for main pulmonary artery diameter (MPAD) measurement (red lines in **c**, **g**), and 3D volumetric reconstruction of pulmonary arteries (**d**, **h**) in a survivor (top) and a non-survivor (bottom) COVID-19 patient, both males of 74 and 76 years, respectively, both with history of hypertension and treated ischemic cardiomyopathy, both suffering from fever (> 37.5 °C) and caught from 7 days. At the admission, WBC/mm^3^ were 4.36 and 3.8, CRP 0.93 and 1.1 mg/dL, LDH 295 and 378 U/L, and creatinine 0.93 and 1.1 mg/dL in survivor and non-survivor, respectively. The oxygen saturation was 90% and 94% for survivor and non-survivor, respectively, with higher well-aerated lung volume in the survivor patient (3882 mL vs 2382 mL, violet parenchyma on 3D lung volume rendering in **a**, **b**, **e**, **f**) with pneumonia (bright parenchyma in **a**, **b**, **e**, **f**), involving from 25 to 50% of lung volume in both cases. Pulmonary artery diameter was normal (27 mm) in the survivor (**c**, **d**) while it was enlarged (32 mm) in the non-survivor (**g**, **h**) who died 9 days after hospital admission

Moderate-severe MPAD enlargement remained associated with a higher risk of death at multiple Cox’s regression analysis also when pneumonia extension, well-aerated lung volume, laboratory tests, demographics, and comorbidities were considered (HR [95% CI]: 1.592 [1.154–2.196], *p* = 0.005) (Table 3). Figure 4 reports two exemplifying cases with normally and moderately enlarged MPAD, respectively. Besides MPAD, the other variables associated with higher risk of mortality in the model were old age (HR [95% CI]: 1.063 [1.052–1.075], *p* < 0.001), male gender (1.586 [1.079–2.329], *p* = 0.019), higher creatinine (2.468 [1.824–3.339], *p* < 0.001), and greater pneumonia score (1.397 [1.084–1.799], *p* = 0.010), while greater well-aerated lung volume was a protective factor (0.699 [0.581–0.841], *p* < 0.001). The final model showed a good calibration (Figure S1) and discrimination (c-index [95% CI]: 0.826 [95% CI: 0.796–0.851]).

**Table 3:** Cox’s multivariable regression model for predicting overall survival estimated in the derivation cohort (c-index [95% CI] = 0.826 [0.796–0.851]; baseline survival at 28 days = 99.92%)

	Derivation cohort (*n* = 761)		
Variable	Coefficient	HR [95% CI]	*p* value
Age, years	0.061	1.063 [1.052–1.075]	< 0.001
Male	0.461	1.586 [1.079–2.329]	0.019
Creatinine, mg/dL	0.903	2.468 [1.824–3.339]	< 0.001
Pneumonia score, %	0.334	1.397 [1.084–1.799]	0.010
Well-aerated lung volume, dm^3^	−0.358	0.699 [0.581–0.841]	< 0.001
Moderate-severe main pulmonary artery diameter enlargement (≥ 31 mm)	0.465	1.592 [1.154–2.196]	0.005

An interaction term between MPAD and gender was included in the final multiple Cox regression model and resulted not to be significant (*p* = 0.724), thus supporting that MPAD has a similar role in both sexes.

### Validation of enlarged MPAD as a predictor of mortality

In the validation cohort, the median age was 69 years (IQR, 59–77 years) and 421 (66.5%) patients were males (Table 1). Hypertension was the most frequent comorbidity (285 patients, 45%) and median oxygen saturation was 92% (IQR, 88–96%).

Most of the patients (626, 98.9%) had pneumonia at chest CT, with prevalent GGOs (328 patients, 51.8%). Thirty-one percent of cases (196) had lung involvement greater than 50%.

Mild, moderate, and severe MPAD enlargement was found in 58 (9.2%), 101 (15.9%), and 33 (5.2%) patients, respectively.

Inter-reader agreement was excellent for both MPAD (ICC [95% CI]: 0.937 [0.931–1]) and pneumonia score (Cohen’s kappa [95% CI]: 0.886 [0.856–0.916]).

The mortality rate of the validation cohort was 22.1% (140/633 patients). OS at 40 days was 77.69% (95% CI = 74.50–81.03%).

At Cox’s regression analysis adjusted with respect to demographics and comorbidities, the risk of mortality associated with moderate-severe MPAD enlargement was slightly higher than in the derivation cohort (HR [95% CI]: 2.524 [1.784–3.571], *p* < 0.001). The discrimination of the model obtained in the derivation cohort was confirmed, using CT measurement from both the first and second reader (c-index [95% CI]: 0.789 [0.758–0.823] and 0.790 [0.753–0.825], respectively).

## Discussion

The main finding of our study is that moderate-severe enlargement of the pulmonary artery diameter (MPAD ≥ 31 mm) at chest CT performed within 72 h from the admission is an independent predictor of death in COVID-19 patients. The multivariable model, coming from the analysis of 761 consecutive COVID-19 patients admitted to three third-level hospitals in Northern Italy, was confirmed on an external validation cohort including 633 patients from four other third-level hospitals in the same Italian territory.

Enlargement of MPAD was associated with a higher rate of in-hospital mortality: 19.4% (105/541) in patients with normal MPAD and 25.6% (22/86), 38.8% (38/98), and 47.2% (17/36) in patients with mild, moderate, and severe MPAD enlargement (global Fisher’s exact test *p* < 0.001).

Enlarged pulmonary artery on chest CT scan was previously demonstrated to be associated with pulmonary hypertension in other clinical settings before COVID-19 outbreak [12, 13, 19]; it is considered a consequence of elevated pressure, reflecting disease severity [13] and duration [20].

MPAD ≥ 31 mm was previously found to have excellent specificity (from 80 to 98%) and positive predictive value (from 85 to 98%) for the diagnosis of PH [13], with a 2–3-fold increased risk of mortality than in patients with normal MPAD [13].

Our results, based on a single time-point CT examination, do not allow to assert if the enlargement of MPAD is acute or not. However, the statistical adjustment for demographics and comorbidities suggested that the observed phenomenon is more likely an acute complication of COVID-19 pneumonia. Moreover, a previous CT study [21] on a limited sample size (44 COVID-19 patients) showed an MPAD enlargement with respect to a previous CT examination in non-survivors, further supporting the hypothesis that MPAD enlargement occurred in response to increased peripheral resistance for COVID-19-associated lung endothelial injury [3, 22, 23].

In the setting of non-COVID-19-related acute respiratory distress syndrome [24], PH resulted in highly prevalent (almost 50%) and entails higher risk of cardiac failure, shock [25, 26], and mortality [26, 27]. The etiology of acute PH is considered to be multifactorial, being related to the refractory hypoxemia, vasoconstriction, pulmonary edema, and microvascular thrombosis [28].

In the COVID-19 setting, where angiotensin-converting enzyme 2 on epithelial cells was recognized as a functional receptor for SARS-CoV-2, low levels of angiotensin and decreased downregulation of angiotensin II may contribute to determine acute PH by increasing pulmonary vasoconstriction [29]. Moreover, pulmonary small-vessel thrombosis could be the main cause of PH in COVID-19 pneumonia. In the autopsy series of COVID-19 patients [1, 2, 30], hallmarks of classic ARDS were not prominent [30], while a pauci-inflammatory thrombogenic vasculopathy [30], with small-vessel thrombosis [1, 2, 23], was reported as a common finding. In our cohort, similar to previous studies [4, 31], higher levels of IL-6, CRP, LDH, D-dimer, and troponin were found in non-survivors compared to survivors, further supporting the hypothesis of microvascular COVID-19 lung thrombo-inflammatory syndrome [4].

The measurement of the MPAD on chest CT is easy, fast (few seconds), highly reproducible, does not require any dedicated software, and could be a useful quantitative marker to be integrated into models for risk stratification. Moreover, the detection of enlarged MPAD on chest CT, being a known sign of PH, may have a potential impact on patient management and treatment. In fact, patients with PH may rapidly deteriorate with RV dysfunction and worsening oxygenation for ventilation-perfusion mismatch. In patients with severe pneumonia and dilated MPAD, alveolar ventilation could be optimized to limit hypoxic vasoconstriction, and prone ventilation preferred for minimizing positive end-expiratory pressure and hemodynamic impact [7]. Close monitoring of pulmonary vascular resistance should be considered to optimize inotropic support and pulmonary vasodilatation [7, 32], decreasing RV afterload.

The limitation of the present study was the lack of data about pulmonary artery pressure estimated by right heart catheterization or echocardiography. Despite echocardiography being commonly used to investigate pulmonary hypertension, it was not routinely performed in all COVID-19 patients. Moreover, its diagnostic accuracy is affected by body habitus, heart rate, acoustic window, operator’s experience [33], and notably by the presence of significant lung disease [5].

In our study, no differences in orotracheal intubation rate were found between patients with normal or enlarged MPAD, despite numerically higher rate in severely enlarged MPAD. This result might be influenced by bias of treatment in overwhelmed hospitals closely related to the scarcity of resources.

Non-survivor rates in our derivation and validation cohorts (23.9% and 22.1%, respectively) are consistent with the mortality rate recorded in Italy during the COVID-19 outbreak [34], suggesting that our cohort could be considered a representative sample of the Italian COVID-19 population.

In conclusion, in the present study, on a large cohort of COVID-19 patients from seven third-level hospitals from Northern Italy, we found that enlarged MPAD measured on chest CT at admission is an independent predictor of mortality. The MPAD can be easily measured on chest CT performed for pneumonia extension evaluation, offering additional prognostic information, and helping clinicians in patients’ management.

## Supplementary information

ESM 1 (DOCX 139 kb)

## Abbreviations

ARDS: Acute respiratory distress syndrome
CRF: Case report form
CRP: C-reactive protein
GGOs: Ground-glass opacities
LDH: Lactate dehydrogenase
LPAD: Left pulmonary artery diameter
MPAD: Main pulmonary artery diameter
PH: Pulmonary hypertension
RPAD: Right pulmonary artery diameter
RT-PCR: Reverse transcriptase polymerase chain reaction
SARS-CoV-2: Severe acute respiratory syndrome coronavirus 2
