# Supplementary material for: Chest CT–derived pulmonary artery enlargement at the admission predicts overall survival in COVID-19 patients: insight from 1461 consecutive patients in Italy
Source: Eur Radiol. 2020 Dec 23;31(6):4031–41. doi: 10.1007/s00330-020-07622-x (PMC7755582; doi:10.1007/s00330-020-07622-x)
Supplement: Supplementary file 1 — (DOCX 139 kb) [file 330_2020_7622_MOESM1_ESM.docx]

**ELECTRONIC SUPPLEMENTARY MATERIALS 1**

**CT scanning protocol**

Chest CT scan were performed in seven third level hospital on 14 multidetector scanners with detector rows from 16 to 256:

- Philips Medical System: Philips MX 16, Philips Brilliance 64, Philips Incisive 128, Philips Ingenuity 128, ICT Elite 256
- GE Medical System: BrightSpeed Elite 16, GE Light Speed VCT 64, GE Optima CT660 – 64, GE Revolution EVO 128, GE Revolution HD 256
- SIEMENS: SOMATOM Emotion 16, SOMATOM Definition AS 64, SOMATOM Definition AS 128
- TOSHIBA: Aquilion 64

CT protocol consisted in a standard ungated chest CT with 80-140 kV, from 0.625 to 2.5 mm of slice thickness and 512 x 512 matrix (DLP: 364 ± 155 mGy cm, mean ± standard deviation).

All chest CT images were reformatted in the core-lab using Matlab R2015a (Mathworks) in order to reach the same slice thickness (2.5 mm) for all the CT scan.

**Statistical Analysis**

Comparisons between numerical variables were performed with Mann-Whitney test, while comparisons between categorical variables with Fisher’s exact test. P-values were adjusted with Bonferroni’s correction to account for multiple testing. Follow-up data were censored at 40 days from hospital admission. Overall survival curve was estimated with the Kaplan-Meier estimator and the log-rank test was applied for comparing groups. The cumulative incidence of oro-tracheal intubation was estimated using the competing risk approach (with death without intubation as competing event) and Gray’s test was performed for comparing groups.

Cox’s proportional hazards regression analysis was employed for evaluating the role of the several classes enlargedof MPAD [1-3] vs normal MPAD in predicting the overall survival, both un-adjusting and adjusting for demographics and

comorbidities (age, sex, hypertension, diabetes, history of cardiovascular disease and chronic lung disease). Based on the results of this analysis, some classes of MPAD were grouped. In order to reduce the effect potential confounding factors when evaluating the difference in the overall survival with respect to the categorized MPAD when using the Kaplan-Meier curves and the log-rank test, adjusted Kaplan-Meier curves and adjusted log-rank test were computed by using the inverse probability weighting with average treatment effect weights based on a propensity score (PS). The PS was computed using a logistic regression model without variable selection. The variables included in the model were the same used for adjusting the Cox’s regression univariate analysis. Multivariable Cox’s proportional hazards regression analysis was employed for

evaluating the simultaneous role of CT parameters (categorized MPAD, pneumonia extension, well

aerated lung volume in dm3), laboratory tests (WBC, CRP, creatinine), demographics and comorbidities (age, sex, hypertension, diabetes, history of cardiovascular disease and chronic lung disease) in predicting the overall survival. The final model was obtained with a backward variable selection of all variables included in the model with removal significant level 0.05. Model calibration was assessed evaluating the curves of agreement between predicted and observed survival at time 28 days, across deciles of predicted survival corresponding to intervals including 50 observations (also optimism corrected curve was computed, since the computation is done on the derivation cohort).

Model discrimination was evaluated with the optimism corrected c-index (since computed on the

derivation cohort) with confidence interval computed using 500 bootstrap resamples. To assess the

role of gender in categorized MAD with respect to the outcome, the final Cox’s model was

estimated adding an interaction term between the two variables.

For validating the role of categorized MPAD as risk factor for overall survival, the adjusted Cox’s regression analysis, the calibration curve and the c-index (with bootstrap confidence interval) of the final multivariable Cox’s model (obtained in the derivation cohort) were computed on the validation cohort.

Rothery’s non-parametric intra-class correlation coefficient (ICC) [4] was computed to assess inter-observer reliability between two readers for the MPAD, while Cohen's kappa coefficient for the pneumonia score.

Missing data were not imputed, thus each analysis considered only complete cases for the variables used in the analysis. All tests were 2-sided and the significance level was set at 0.05. Confidence intervals (CIs) were computed at 95% level. All statistical analyses were performed using R 3.5.0 (<http://www.R-project.org/>). The survival package was employed for performing survival analysis, IPW survival for adjusted survival analysis with inversed probability weighting, survminer for plotting survival curves, cmprsk for performing competing risk analysis, the rms package for computing the calibration curve and c-index, the psych package for the Cohen's kappa while the nopaco package for the Rothery’s ICC.

References

1. Truong QA, Bhatia HS, Szymonifka J, et al. A four-tier classification system of pulmonary artery

metrics on computed tomography for the diagnosis and prognosis of pulmonary hypertension. *J*

*Cardiovasc Comput Tomogr.* 2018;12(1):60-66.

2. Tan RT, Kuzo R, Goodman LR, Siegel R, Haasler GB, Presberg KW. Utility of CT scan

evaluation for predicting pulmonary hypertension in patients with parenchymal lung disease.

Medical College of Wisconsin Lung Transplant Group. Chest. 1998;113(5):1250-1256.

3.Truong QA, Massaro JM, Rogers IS, et al. Reference values for normal pulmonary artery

dimensions by noncontrast cardiac computed tomography: the Framingham Heart Study. *Circ*

*Cardiovasc Imaging.* 2012;5(1):147-15.

4. Rothery (1979). A nonparametric measure of intraclass correlation, Biometrika 66(3):629-639.

**Figure S1 Calibration analysis of the final multivariable Cox’s regression model in both the derivation and validation cohorts.**

For each cohort, the curves represent predicted versus observed survival at time 28 days, across deciles of predicted survival corresponding to intervals including 50 observations. The curves show a good agreement. The grey line represents the ideal curve, the black line the observed curve, while the blue line the optimism-corrected curve (obtained with 500 bootstrap samples).


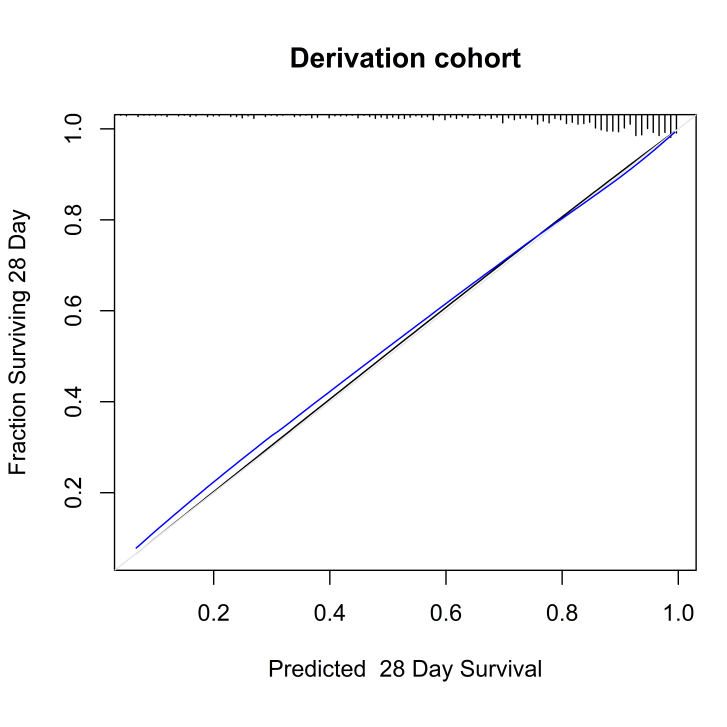

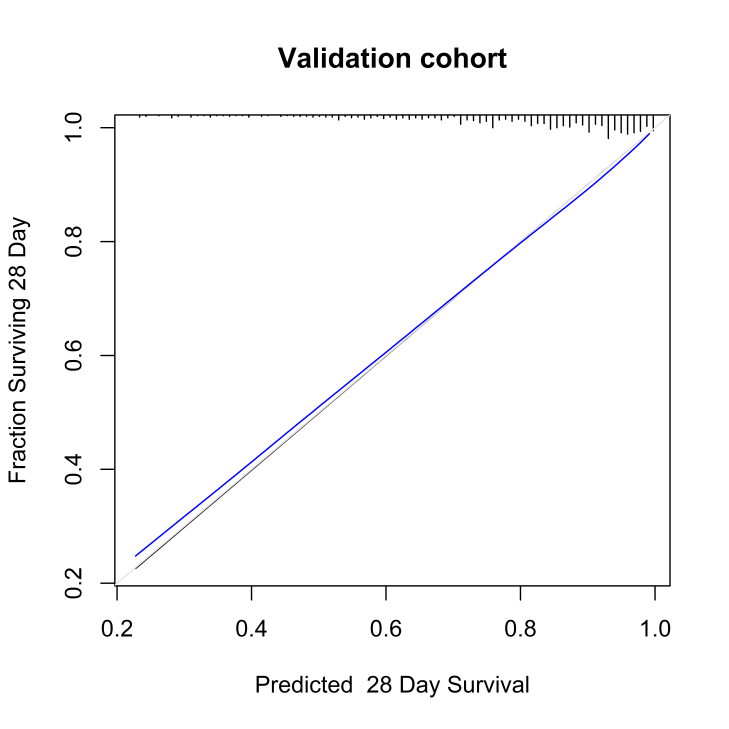


**Table S1.** Cox’s univariate regression model for evaluating the role of the several classes of enlarged MPAD vs normal MPAD in predicting the overall survival in the derivation cohort.

|  | **Derivation cohort (n=761)** | |
| --- | --- | --- |
| **Variable** | **HR [95% CI]** | **p-value** |
| Main pulmonary artery diameter enlargement |  |  |
| mild vs normal | 1.387 [0.876-2.196] | 0.163 |
| moderate vs normal | 2.311 [1.594-3.350] | <0.001 |
| severe vs normal | 3.001 [1.797-5.010] | <0.001 |

**Table S2**. Main clinical and CT features of females vs males in the derivation cohort.

|  | **Females**  **(n=224)** | **Males**  **(n=537)** | |  |
| --- | --- | --- | --- | --- |
| **variables** | **median [IQR]** | **median [IQR]** | **adj. p-value** | |
| Age, y | 70.83 [60.35-77.82] | 68.62 [57.34-76.64] | 0.515 | |
| Hypertension, No. (%) | 151 (67.4%) | 333 (62%) | 1.000 | |
| Diabetes, No. (%) | 35 (15.6%) | 115 (21.4%) | 0.939 | |
| History of cardiovascular disease, No. (%) | 17 (7.6%) | 78 (14.5%) | 0.103 | |
| History of chronic lung disease, No. (%) | 33 (14.7%) | 62 (11.5%) | 1.000 | |
| White Blood Cells Count/mm3 | 6350 [4632.5-9280] | 6740 [5100-9692.5] | 1.000 | |
| Creatinine, mg/dL | 0.8 [0.67-1.04] | 1.09 [0.92-1.35] | <0.001 | |
| CRP, mg/dL | 9.13 [3.66-16.3] | 12.08 [6.43-20.06] | <0.001 | |
| Main pulmonary diameter, No. (%) |  |  | 0.002 | |
| normal | 147 (65.6%) | 394 (73.4%) |  | |
| mild enlarged | 43 (19.2%) | 43 (8%) |  | |
| moderate enlarged | 28 (12.5%) | 70 (13%) |  | |
| severe enlarged | 6 (2.7%) | 30 (5.6%) |  | |
| Moderate-severe main pulmonary artery diameter enlargement (≥31 mm) | 34 (15.2%) | 100 (18.6%) | 1.000 | |
| Well-aerated lung volume, cm^3^ | 1943 [1182.25-2966.25] | 2493 [1472-3580] | <0.001 | |
| Pneumonia score, No. (%) |  |  | 0.002 | |
| 0 | 6 (2.7%) | 3 (0.6%) |  | |
| 1 | 96 (42.9%) | 154 (28.7%) |  | |
| 2 | 84 (37.5%) | 279 (52%) |  | |
| 3 | 36 (16.1%) | 94 (17.5%) |  | |
| 4 | 2 (0.9%) | 7 (1.3%) |  | |
| Non-survivors (40-days FU), No. (%) | 41 (18.3%) | 141 (26.3%) | 0.257 | |
